# Analytical study of the spherical hydrostatic bearing dynamics through a unique technique

**DOI:** 10.1038/s41598-023-46296-5

**Published:** 2023-11-08

**Authors:** Ahmad Waguih Elescandarany

**Affiliations:** Faculty of Engineering, Alexandria University, Lotfy el Sayed Street, Ebrahimia, Alexandria, Egypt

**Keywords:** Engineering, Mathematics and computing

## Abstract

Because of their self-alignment property and design simplicity, the spherical hydrostatic bearings have the advantage over the other bearing configurations. Their static and dynamic performances have been intensively studied. Focusing on the bearing dynamic performance, it could be realized that the researchers used to mechanically excite the bearing in the experimental studies and perturb the rotor (spatial finite displacement) in the theoretical studies, observing its behavior and expressing it by dynamic stiffness and damping coefficient. Owing to a lack of information on bearing oscillation, this study adopts a new method to analyze this bearing behavior theoretically and grasp its nature. Unusually, the bearing vibration is studied hydro-dynamically rather than mechanically, showing the effect of eccentricity, inertia, restrictor type, seat configuration, and the supply pressure on the performance. New and unique formulas have been derived to predict the frequency, stiffness, and dampness, explaining how these parameters create the bearing self-alignment property. Some thrust bearings, designed with different techniques, have been examined where the results show vibration even in the bearing’s stationary state. Also, the newly derived formulas could precisely predict the rotation and rotational speed of the Kugel ball (recently proved and designed as a spherical hydrostatic bearing).

## Introduction

Bearings constitute a fundamental component of any rotating machine, where a good understanding of their behavior allows the prediction of the system function. High speeds and high power with reliable operation are necessary. Accurate prediction and control of bearing behavior is required. It is commonly known that more than 40% of machine failures are the result of bearing problems. The most efficient approach to solving the problem is through analysis conducted in numerous studies, which leads to better performance. The potential characteristics of low running friction, small viscous dissipation, high load carrying capacity, and high stiffness have made hydrostatic bearings indispensable for industrial applications in present-day heavy-duty and high-speed devices such as machine tools, precision measuring instruments, hydraulic piston pumps, motors, telescopes, gyroscope gimbals, dynamometers, radar tracking units, and craft engines. Spherical bearings have advantages over other configurations due to their self-alignment property and their capacity to accept radial and thrust loads. Therefore, the operation of the spherical bearings is not affected by angular misalignments. Mayer^1^ and Elescandarany^2^ derived the Reynolds governing equation for spherical bearing applications. Kazama^3^ presented an optimal design for the types of fitted and clearance thrust spherical bearings with capillary and orifice restrictors. This design was based on minimizing power losses and maximizing stiffness. The study concluded that the central pressure ratio is two-thirds for the fitted type and 0.69 for the clearance type. Yacout^4–8^ analytically studied both types after developing the Reynolds equation to apply it to the thrust spherical bearing, considering the effects of surface roughness, centripetal inertia, viscosity, and seat configuration on bearing performance. The central pressure ratio was calculated to be two-thirds for both types, leading to consistent bearing performance. Examples of optimal designs based on minimum friction and flow rate were provided. Elescandarany^9–11^ developed two new techniques to design a fitted type, with and without a restrictor. Based on the steady and unsteady states, Gosh et al.^12–14^ and San Ander^15^ numerically studied the dynamic behavior of the journal bearing. This behavior is represented through stiffness and damping coefficients by exciting the bearing journal while also considering the fluid compressibility in the bearing recess. Applying the first-order perturbation method, Yacout^16, 17^ studied the dynamic behavior (dynamic stiffness and damping coefficient) of the thrust spherical bearing, considering the compressibility of the fluid in the bearing recess. Sharma et al.^18, 19^ numerically studied thrust and journal spherical bearings with Newtonian and non-Newtonian fluids, considering the effect of surface roughness on bearing performance. Gupta and Kumar^20, 21^ examined the behavior of a hydrodynamic squeeze film between a stationary spherical surface and a hemispherical bearing. The research focused on understanding the impact of surface roughness on bearing performance when subjected to a constant load. Based on self-excited vibration, the studies generally are concerned with techniques to control and suppress the vibration. Cameron^22^ stated that one of the most troublesome features of high-speed bearings could be oil whirl. It is a vibration that occurs slightly below half the speed of the shaft and is usually found during acceptance trials, which is the most awkward time for serious faults to appear. The main features of the problem were analyzed and understood a few years ago. There are now a few guidelines for the solution to the problem. A bearing that completely suppresses oil whirl has not yet been produced, though new anti-whirl bearing configurations are patented yearly. Rowe^23^ addressed the whirl phenomenon, stating that the puzzling question is the following: How is it possible for a heavily damped system to act as though it has zero dampness, and replying that the explanation is not altogether simple and lies like the hydrodynamic stiffness causing a positive reaction force in the direction of the velocity, in turn, this force acts in opposition to the squeeze force and cancels out the dampness. Jinhao et al.^24^ presented an experimental study on the active control of self-excited vibrations in a rotor bearing system supported on a pair of externally pressurized thrust bearings in the axial direction and on actively controlled journal gas bearings in the radial direction. Feedback control systems were constructed with gap sensors to measure the vibration of the piezoelectric actuators embedded in the rotor and thee PID (proportional–integral–derivative) controllers. The experimental results show that self-excited vibration could be effectively suppressed with the designed feedback control system if the gains of the PID controllers are appropriately tuned. An experimental study offered by Talukder and Stowell^25^ was made of the occurrence of a pneumatic hammer in an externally pressurized air journal with admission into double plane. Various parameters that affect the onset of the pneumatic hammer were investigated, such as the depth of the recess, the diameter of the orifice and the bearing mass, stating that the pneumatic hammer is relatively easily avoided in the journal bearings. Taiwari et al.^26^ presented research reviewing the bearings' dynamic parameters in rotating machines. Attention is given to vibration-based methods where the review covers descriptions of experimental measurement techniques, mathematical modeling, parameter extraction algorithms, and uncertainty in the estimates applied to a variety of bearings and suggests some articles as the need for more experimental work in the field of rotor dynamics to study the bearing and support influence upon the rotor response, especially for full-scale rotor systems. Ales Tondl^27^ presented a survey of publications dealing with vibration suppression methods in different types of self-excitation. The attention is given to passive and active means using parametric excitation, focusing on the latter, and the necessary steps for answering the main questions are formulated to initiate further investigation, especially application to actual systems. Feng and Hahn^28^ presented a study on circular and elliptical bearings to examine and compare their impact on the rotor support of industrial machines. The findings showed significant variations in natural frequencies and critical speeds depending on the type of bearing used. Charki et al.^29^ provided a numerical simulation and an experimental study to assess the stiffness and dampness characteristics of thrust air bearings with multiple orifices using finite element modeling to solve the Reynolds equation. The results obtained numerically showed that performance is related to bearing design, and the experimental investigation allowed analyzing bearing behavior. Peiji et al.^30^ studied the impacts of cylindrical bearings and four-lobe bearings on the vibration of the rotor system, combining theoretical analysis and experimental results, concluding that different types of bearing have effects on the critical characteristics of the rotor system and the vibration of the rotor supported by the cylindrical bearing is more stable than the four-lobe bearing, where the results showed good agreement between theoretical and experimental analysis. Zhifeng et al.^31^ presented a review of research articles related to the introduction of developments in hydrostatic bearings (the basic theory contains equations and analysis methods including analytic, numerical, and experimental methods) where typical applications were based on rectangular oil pads, circular oil pads, and journal bearings. Polach et al.^32^ presented a simulation for a system consisting of a rotor supported on two journal bearings and a controller, which controls the speed of the rotor, to study self-excited and flow-induced vibrations of the rotor, reporting that subsynchronous flow-induced vibrations can occur only if the rotor is well balanced and is radially supported on poorly lubricated journal bearings, whose supply bores are located in the lower bearings half. Davorka and Marko^33^ presented a modern method for testing the condition of bearings in operation using vibration analysis and demonstrating the importance of this method by changing the parameters related to two different bearing structures grouped in the so-called Sommerfeld number. Dmytro et al.^34^ presented a study to improve machining accuracy in precision cutting and grinding by designing a developed spindle with water-lubricated hydrostatic bearings. Dynamic characteristics such as stiffness, dampness, and frequency of the machine spindle were investigated by simulation and experiments, where it is reported that the simulation results were in good agreement with the actual spindle dynamics obtained experimentally. Yingjie Wang et al.^35^ studied the characteristics of hydrostatic bearings by analyzing load capacity, stiffness, and dampness using CFD and 3D meshing, hence Compared to traditional empirical formulae, 3D meshing and CFD analysis were more accurate and could be adapted to the analysis of various shapes of hydrostatic bearings. Runlin Chen et al.^36^ studied a motorized hydrostatic spindle system of a grinding machine using a two-degree-of-freedom stiffness model, considering the rotation of the rotor, where stiffness was analyzed under different excitation frequencies, and the vibration test in the spindle system was implemented through the hammering method. The stiffness of the spindle system was found to agree well with the theoretical calculation, with an average error of about 14%. Xin Qin et al.^37^ presented a novel hydrostatic squeeze film metal mesh journal bearing, which uses both a hydrostatic squeeze film damper and a metal mesh damper to suppress vibration of rotor bearing systems where lubrication equations were introduced to calculate the dynamic characteristics. Experiments were conducted to study the reduction in vibration of the bearing rotor system with the bearing; the theoretical and experimental results showed that the bearings exhibit excellent dampness and vibration attenuation characteristics. Despite the attention given to the journal bearing through numerous studies handling its static and dynamic behavior, in addition to the recent studies on its self-excitation vibration and endless research on eliminating, suppressing, and controlling this undesired disturbance by active or passive means, nothing has been offered about the vibratory behavior or/and the self-alignment property of the spherical hydrostatic type of bearings. Therefore, as the next step to designing an ideal model of such a bearing, this paper presents a new study investigating the natural properties of this bearing by applying a new method depending on the Reynolds equation and the hydrodynamics of the bearing.

## Theoretical analysis

From^1, 4, 16, 17^:

### Previously derived relations

**Figure 1:**
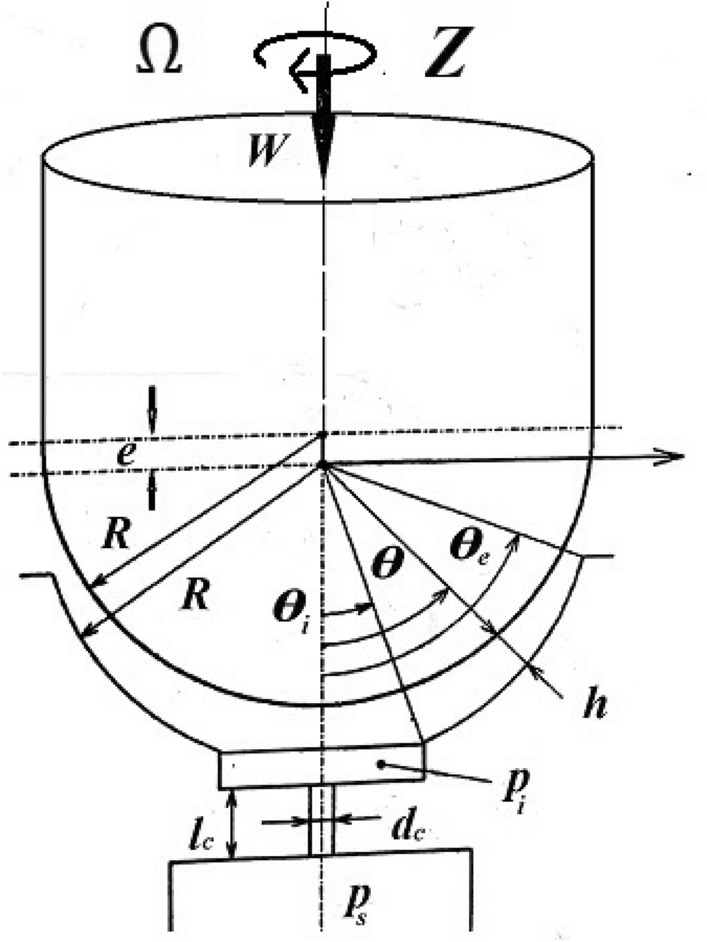
Bearing configuration.

From Yacout^4^:

The configuration of the bearing, Fig. 1, and its related formulas are set below.

1 $$\begin{gathered} h = \,e\,\cos \,\theta \quad (a) \hfill \\ \theta_{e} \cong \varphi _{b} \, = \frac{\pi }{2}\eta \quad (b) \hfill \\ \beta = \frac{2}{3}\quad (c) \hfill \\ q = K_{c} \frac{{p_{s} }}{3\,\mu }\quad (d) \hfill \\ q = K_{o} \sqrt {\frac{{p_{s} }}{3}} \quad (e) \hfill \\ \end{gathered}$$

### Governing equations

From^1, 4, 16, 17^:

2 $$\frac{d\,}{{d\theta }}\,\left[ {h^{3} \sin \theta \frac{{d\mathop p\limits^{ - - } }}{d\theta }} \right] = 12\,\mu \,R^{2} \,\sin \theta \frac{dh}{{dt}}$$

3 $$\left[ {h^{3} \sin \theta \frac{{d\mathop p\limits^{ - - } }}{d\theta }} \right] = C + \left( {\frac{{3\,\rho \,\Omega^{2} \,R^{2} }}{20}} \right)\,h^{3} \,\sin \theta \,\sin 2\theta$$

## Mathematics

The following is a unique method to derive new mathematical expressions that predict the bearing performance and elucidate its characteristics.

### Derivation of mathematical expressions

Arranging and integrating Eq. (2) give:

4 $$\left[ {h^{3} \sin \theta \frac{{d\mathop p\limits^{ - - } }}{d\theta }} \right] = 12\,\mu \,R^{2} \,\int\limits_{0}^{{\theta_{e} }} {\sin \,\theta \frac{dh}{{dt}}} d\theta$$

Differentiation of Eq. (1a) (w. r. t) time gives:

5 $$\frac{dh}{{dt}} = \,\frac{de}{{dt}}\cos \,\theta - e\,\sin \,\theta \frac{d\theta }{{dt}}$$

### Relation between ($\mathop \theta \limits^{ - }$) and the frequency (***f***)

Equation (1b) could be put in the form:

$$\theta_{e} \left( {\frac{4}{{t_{o} }}} \right) = \,\left( {\frac{4}{{t_{o} }}} \right)\frac{2\pi }{4}\eta$$

Hence:

$$\begin{gathered} \frac{{\theta_{e} }}{{\left( {\frac{{t_{o} }}{4}} \right)}} = 4\,\frac{2\pi }{{4t_{o} }}\eta \hfill \\ \frac{{\theta_{e} }}{t} = 4\,\frac{2\pi }{T}\eta \hfill \\ \end{gathered}$$

Then:

6 $$\begin{gathered} \overline{\theta } = 4\,\omega \eta \hfill \\ \overline{\theta } = 8\pi \,\eta \,f \hfill \\ \frac{t}{T} = \frac{1}{16} \hfill \\ \end{gathered}$$

### Bearing vibration parameters

#### The frequency

From Eq. (5) and for steady state:

$$\begin{gathered} \overline{h}\, = \,0 \hfill \\ \overline{e}\,\, = \,e\overline{\theta }\,\tan \theta \hfill \\ \end{gathered}$$

And because of the inversely proportional relation between (e) and ($\theta$) then:

7 $$\overline{e}\, = \, - e\overline{\theta }\,\tan \theta$$

From Eq. (5) in to Eq. (4):

8 $$\left[ {h^{3} \sin \theta \,\,\frac{{d\mathop p\limits^{ - - } }}{d\theta }} \right] = 12\,\mu \,R^{2} \,\int\limits_{0}^{{\theta_{e} }} {\left[ {\overline{e}\,\,\sin \theta \cos \,\theta - \,e\,\overline{\theta }\sin^{2} \theta } \right]d\theta }$$

The integration of Eq. (8) gives:

9 $$\left[ {h^{3} \sin \theta \,\,\frac{{d\mathop p\limits^{ - - } }}{d\theta }} \right] = 6\,\mu \,R^{2} \,\left[ {\overline{e}\,\,\sin^{2} \theta_{e} - \,e\,\overline{\theta }\,(\theta_{e} - \sin \theta_{e} \,\cos \theta_{e} )} \right]$$

From Eqs. (6, 7) in to Eq. (9):

10 $$\left[ {h^{3} \sin \theta \,\,\frac{{d\,\mathop p\limits^{ - - } }}{d\theta }} \right] = 48\pi \,\mu \,R^{2} e\,\eta \,f\,\,\left[ {(\theta_{e} - \sin^{2} \theta_{e} \,(\cot \theta_{e} \, - \tan \theta )} \right]$$

From Eqs. (3, 10):

$$C + \left( {\frac{{3\,\rho \,\Omega^{2} \,R^{2} }}{20}} \right)\,h^{3} \,\sin \theta \,\sin 2\theta \, = 48\pi \,\mu \,R^{2} e\,\eta \,f\,\,\left[ {(\theta_{e} - \sin^{2} \theta_{e} \,(\cot \theta_{e} \, - \tan \theta )} \right]$$

11 $$\begin{gathered} f = \frac{1}{{48\pi \,\mu \,R^{2} e\,\eta }}\left| {\frac{{C + \left( {\frac{{3\,\rho \,\Omega^{2} \,R^{2} }}{20}} \right)\,h^{3} \,\sin \theta \,\sin 2\theta }}{{\theta_{e} - \sin^{2} \theta_{e} \,(\cot \theta_{e} \, - \tan \theta )}}} \right| \hfill \\ or \hfill \\ f = \frac{1}{{48\,\mu \,e\,A_{s} \,\eta }}\left| {\frac{{C + S_{1} \,h^{3} \,\sin \theta \,\sin 2\theta }}{{\theta_{e} - \sin^{2} \theta_{e} \,(\cot \theta_{e} \, - \tan \theta )}}} \right| \hfill \\ S_{1} = 3\,\rho \,\Omega^{2} \,R^{2} /20 \hfill \\ A_{s} = \pi \,R^{2} \hfill \\ \end{gathered}$$

From the Appendix:

12 $$\begin{gathered} C = Q\,e^{3} \,\beta \,p_{s} \hfill \\ f = \frac{1}{{48\,\mu \,e\,A_{s} \,\eta }}\left| {\frac{{Q\,\,e^{3} \,\beta \,p_{s} + S_{1} \,h^{3} \,\sin \theta \,\sin 2\theta \,}}{{\theta_{e} - \sin^{2} \theta_{e} \,(\cot \theta_{e} \, - \tan \theta )}}} \right| \hfill \\ \end{gathered}$$

#### The restrictor effect on the frequency

##### Capillary tube restrictor

From Yacout^4^

$$C = \frac{6\,\mu \,q}{\pi },\;q = \frac{{K_{c} \,p_{s} }}{3\mu },\;K_{c} = \frac{{\pi \,d_{c}^{4} }}{{128\,l_{c} }},\;n_{c} = \frac{{\,\,l_{c} }}{d}\,\, > \,20,\;m_{c} = \frac{{\,\,d_{c} }}{{d_{i} }},\;d_{i} = 2\,R\,\sin \phi_{i}$$

Then:

$$C_{c} = \left( {\frac{{m_{c} \,R\,\sin \phi_{i} }}{2}} \right)^{3} \,\,(p_{s} /n_{c} )$$

Equation (12) becomes:

13 $$\begin{gathered} C = Q\,e^{3} \,\beta \,p_{s} \hfill \\ f_{c} = \frac{1}{{48\,\mu \,e\,A_{s} \,\eta }}* \hfill \\ \left| {\frac{{\left( {\frac{{m_{c} \,R\,\sin \phi_{i} }}{2}} \right)^{3} \;(p_{s} /n_{c} ) + S_{1} \,h^{3} \,\sin \theta \,\sin 2\theta \,}}{{\theta_{e} - \sin^{2} \theta_{e} \,(\cot \theta_{e} \, - \tan \theta )}}} \right| \hfill \\ \end{gathered}$$

##### Orifice restrictor

From Yacout^4^

$$C = \frac{6\,\mu \,q}{\pi },\;q = K_{o} \,\left( {\frac{{\,p_{s} }}{3}} \right)^{1/2} ,\;K_{o} \, = \left( {\frac{{\,\pi \,C_{d} \,d_{o}^{2} }}{{\sqrt {8\,\rho } }}} \right),\;m_{o} = \frac{{\,\,d_{o} }}{{d_{i} }},\;d_{i} = 2\,R\,\sin \phi_{i}$$

Then:

$$C_{o} = 2\,\mu \,C_{d} (m_{o} \,R\,\sin \phi_{i} )^{2} \,\,(6\,p_{s} /\rho )$$

Equation (12) becomes:

14 $$\begin{gathered} C = Q\,e^{3} \,\beta \,p_{s} \hfill \\ f_{o} = \frac{1}{{48\,\mu \,e\,A_{s} \,\eta }}* \hfill \\ \left| {\frac{{2\,\mu \,C_{d} (m_{o} \,R\,\sin \phi_{i} )^{2} \,(6\,p_{s} /\rho ) + S_{1} \,h^{3} \,\sin \theta \,\sin 2\theta \,}}{{\theta_{e} - \sin^{2} \theta_{e} \,(\cot \theta_{e} \, - \tan \theta )}}} \right| \hfill \\ \end{gathered}$$

#### The wave velocity

The wave velocity is ($\overline{e}$).

From Eq. (7):

15 $$\overline{\theta } = - \frac{{\overline{e}}}{e}\,\cot \theta$$

From Eq. (15) in to Eq. (8) and integration give:

16 $$\left[ {h^{3} \sin \theta \,\,\frac{{\partial \,\mathop p\limits^{ - - } }}{\partial \theta }} \right] = 12\,\mu \,R^{2} \,\overline{e}\,\,\sin^{2} \theta_{e}$$

From Eq. (3, 16) then:

$$12\,\mu \,R^{2} \,\overline{e}\,\,\sin^{2} \,\theta_{e} = C + S_{1} h^{3} \,\sin \theta \,\sin 2\theta \,$$

17 $$\overline{e}\,\, = \frac{{C + S_{1} h^{3} \,\sin \theta \,\sin 2\theta }}{{12\,\mu \,R^{2} \,\sin^{2} \,\theta_{e} \,}}\,$$

#### The wave length

In physics:

*Wave velocity = wave length × frequency:*

Hence:

$$\begin{gathered} \overline{e}\, = \,\lambda \,f \hfill \\ \lambda = \overline{e}/f\, = \,\left[ {\frac{{C + S_{1} \,h^{3} \,\sin \theta \,\sin 2\theta \,}}{{12\,\mu \,R^{2} \,\sin^{2} \,\theta_{e} }}\,} \right]/ \hfill \\ \left\{ {\frac{1}{{48\,\mu \,e\,A_{s} \,\eta }}\left| {\frac{{C + S_{1} \,h^{3} \,\sin \theta \,\sin 2\theta \,}}{{\theta_{e} - \sin^{2} \theta_{e} \,(\cot \theta_{e} \, - \tan \theta )}}} \right|} \right\} \hfill \\ \end{gathered}$$

Then:

18 $$\begin{gathered} \lambda = (4\,\pi \,\eta \,e)\,(\alpha + \tan \theta ) \hfill \\ \alpha = \frac{{2\,\theta_{e} - \sin 2\theta_{e} }}{{2\sin^{2} \theta_{e} }} \hfill \\ \end{gathered}$$

#### The wave power

From^38, 39^:

19 $$\,P_{w} = \frac{{\rho \,g^{2} \,H^{2} T}}{6\,4\,\pi }\,$$

### The characteristic equation of the rotor displacement

From Eq. (7):

$$\begin{gathered} - \mathop e\limits^{ = } \, = \,\frac{\partial }{\partial \,t}\,(e\overline{\theta }\,\tan \theta \,) = \hfill \\ e\frac{\partial }{\partial \,t}\,\left( {\overline{\theta }\,\tan \theta } \right) + \overline{\theta }\,\tan \theta \,\frac{\partial \,e}{{\partial \,t}} = \hfill \\ e\left\{ {\left[ {\overline{\theta }\,\frac{\partial }{\partial \,t}\,(\tan \theta ) + \mathop \theta \limits^{ = } \tan \theta } \right]} \right\} + \overline{e}\overline{\theta }\tan \theta = \hfill \\ e\,\left[ {\overline{\theta }\,\,(\overline{\theta }\,\sec^{2} \theta ) + \mathop \theta \limits^{ = } \tan \theta } \right] + \overline{e}\tan \theta = \, \hfill \\ e\left( {\mathop {\theta^{2} }\limits^{ - } \,\sec^{2} \theta + \mathop \theta \limits^{ = } \tan \theta } \right) + \overline{e}(\overline{\theta }\tan \theta ) \hfill \\ \,\mathop e\limits^{ = } + \,\overline{e}\left( {\overline{\theta }\tan \theta } \right) + e\,\left( {\mathop {\theta^{2} }\limits^{ - } \,\sec^{2} \theta + \mathop \theta \limits^{ = } \tan \theta } \right) = 0 \hfill \\ \end{gathered}$$

Then:

20 $$\begin{gathered} \mathop e\limits^{ = } + \,K_{1} \,\overline{e} + K_{2} \,e\,\, = 0 \hfill \\ K_{1} = \left( {\overline{\theta }\tan \theta } \right) \hfill \\ K_{2} = \left( {\mathop {\theta^{2} }\limits^{ - } \,\,\,\sec^{2} \theta + \mathop \theta \limits^{ = } \tan \theta } \right) \hfill \\ \end{gathered}$$

From Eq. (6):

21 $$\begin{aligned} \overline{\theta } = & 8\pi \,\eta \,f\,,\,\,\,t = T/16 \\ \mathop \theta \limits^{ = } = & 8\pi \,\eta \,\frac{d}{dt\,}\,\left( \frac{1}{T} \right) = \,128\pi \,\eta \,\frac{d}{dT}\,\left( \frac{1}{T} \right) \\ = & \, - \frac{128\pi \,\eta }{{T^{2} }} = - 128\pi \,\eta \,f^{2} \\ \mathop \theta \limits^{ = } = & - 128\pi \,\eta \,f^{2} \\ \end{aligned}$$

From Eqs. (6, 21) in to Eq. (20):

22 $$\begin{aligned} \mathop e\limits^{ = } & + \,K_{1} \,\overline{e} + K_{2} \,e\,\, = 0 \\ K_{1} = & (8\,\pi \eta \,f\,\tan \theta ) \\ K_{2} = & (64\,\pi \eta \,8\,\pi \eta \,f^{2} )\,\,(\pi \,\eta \,\sec^{2} \theta - 2\,\,\tan \theta ) \\ \end{aligned}$$

### The characteristic equation solution

Hint:

It would be useful to replace (e) with (E) in the equation to prevent the confusion between (e) the eccentricity and (e) the exponential number and after the solution it would be returned to its original form.

Hence it would be:

$$\mathop E\limits^{ = } + \,K_{1} \overline{E} + K_{2} \,E\,\, = 0$$

Then, the solution is:

$$E = \,a\,e^{{K_{1} t}} + b\,e^{{K_{2} t}}$$

Using the boundary conditions:

$$\begin{gathered} At \hfill \\ t = 0\, \to \,E = E \hfill \\ Then \hfill \\ a = E - b \hfill \\ \end{gathered}$$

$$\begin{gathered} \& \,At \hfill \\ t = T\, \to \,E = \lambda \hfill \\ Then \hfill \\ b = \frac{{E\,e^{{K_{1} T}} - \lambda }}{{e^{{K_{1} T}} - e^{{K_{2} T}} }} \hfill \\ \end{gathered}$$

Returning the equation to its original form will be:

23 $$\begin{gathered} e = a\,e^{{K_{1} t}} + be^{{K_{2} t}} \hfill \\ b = \frac{{e\,e^{{K_{1} T}} - \lambda }}{{e^{{K_{1} T}} - e^{{K_{2} T}} }} \hfill \\ a = e - b \hfill \\ K_{1} = (8\,\pi \eta \,f\,\tan \theta ) \hfill \\ K_{2} = (64\,\pi \eta \,8\,\pi \eta \,f^{2} )\,\,(\pi \,\eta \,\sec^{2} \theta - 2\,\,\tan \theta ) \hfill \\ \end{gathered}$$

### Bearing pressure

Arranging Eq. (3) gives:

$$d\,\mathop p\limits^{ - - } = \left[ {\frac{C}{{h^{3} \sin \theta }} + \left( {\frac{{3\,\rho \,\Omega^{2} \,R^{2} }}{20}} \right)\,\sin 2\theta \,} \right]d\theta$$

The integration could be found in the appendix as:

24 $$\begin{gathered} P = A\,\left[ {\ln (\cot \theta ) - \frac{{\sec^{2} \theta }}{2}} \right] + S_{2} \sin^{2} \theta \, + B \hfill \\ A = \frac{{1 - S_{2} (\sin^{2} \theta_{i} - \,\sin^{2} \theta_{e} )}}{{\ln \left( {\frac{{\cot \theta_{i} }}{{\cot \theta_{e} }}} \right) - \frac{{\sec^{2} \theta_{i} - \sec^{2} \theta_{e} }}{2}}} \hfill \\ B = - A\,\left[ {\ln (\cot \theta_{e} ) - \frac{{\sec^{2} \theta_{e} }}{2}} \right] + S_{2} \sin^{2} \theta_{e} \hfill \\ S_{2} = \left( {\frac{{3\,\rho \,\Omega^{2} \,R^{2} }}{{20p_{i} }}} \right) \hfill \\ \end{gathered}$$

### Bearing load capacity

From^4^:

25 $$\begin{aligned} w = & \pi R^{2} p_{i} \sin^{2} \theta_{i} + 2\pi R^{2} \int\limits_{{\theta_{i} }}^{{\theta_{e} }} {p\sin \theta \cos \theta \,\,d\theta } \\ \frac{w}{{\pi R^{2} p_{i} }} = & \sin^{2} \theta_{i} + 2\int\limits_{{\theta_{i} }}^{{\theta_{e} }} {\frac{p}{{p_{i} }}\sin \theta \cos \theta \,\,d\theta } \\ W = & \sin^{2} \theta_{i} + 2\int\limits_{{\theta_{i} }}^{{\theta_{e} }} {P\sin \theta \cos \theta \,\,d\theta } \\ W = & \sin^{2} \theta_{i} + 2\int\limits_{{\theta_{i} }}^{{\theta_{e} }} {\left[ {A\,\left[ {\ln (\cot \theta ) - \frac{{\sec^{2} \theta }}{2}} \right] + S_{2} \sin^{2} \theta \, + B} \right]\sin \theta \cos \theta \,\,d\theta } \\ \end{aligned}$$

The integration of Eq. (25) could be found in the appendix:

26 $$W = \sin ^{2} \theta _{i} + \left\{ {A\left[ {\sin ^{2} \theta \ln (\cot \theta ) + - \frac{1}{2}} \right] + \left. {\frac{{S_{2} }}{2}\sin ^{4} \theta {\mkern 1mu} + B\sin ^{2} \theta } \right\}_{{\theta _{i} }}^{{\theta _{{ei}} }} } \right\}$$

### The bearing dynamic stiffness

**Figure 2:**
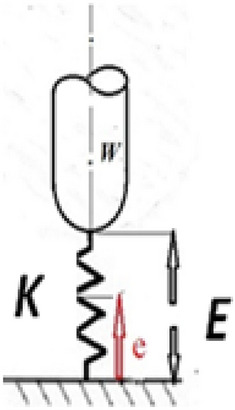
Bearing stiffness configuration.

From Eqs. (23, 26), the dynamic stiffness could be calculated as (Fig. 2):

27 $$\begin{gathered} K = \frac{\,W}{e} \hfill \\ K = \,\frac{W}{{a\,e^{{K_{1} t}} + b\,e^{{K_{2} t}} }} \hfill \\ \end{gathered}$$

### The bearing dampness

The dampness could be calculated as:

28 $$\begin{gathered} D = \,W/\overline{e} \hfill \\ D = \left[ {\frac{{12\,\mu \,R^{2} \,\sin^{2} \,\theta_{e} }}{{C + S_{1} h^{3} \,\sin \theta \,\sin 2\theta }}} \right]\,W \hfill \\ \end{gathered}$$

## Bearing vibration inspection

Rotating machines must be subjected to vibration inspection to evaluate condition and efficiency where defects could be revealed.

### The vibration measurement

Vibration could be measured using one of the following measurement techniques: displacement, velocity or acceleration.

### The measurement position

The probe position on the bearing depends on the measuring technique.

### The vibration remedy

The vibration could be suppressed via passive or active means.

## Results

**Figure 3:**
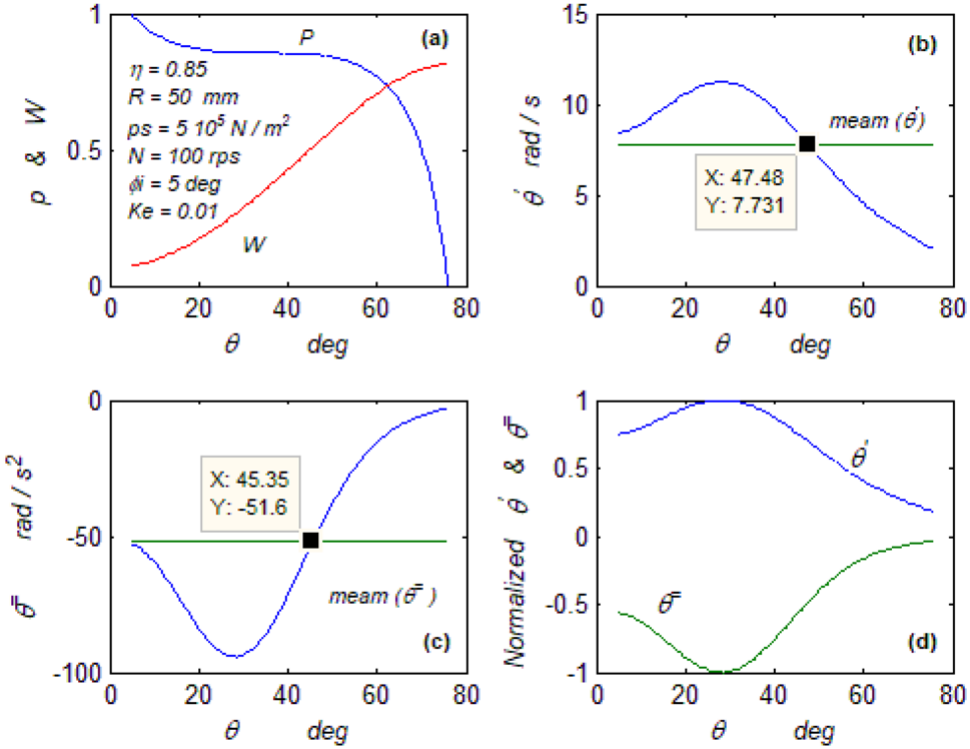
Bearing vibratory factors distribution. (**a**) Local pressure and load, (**b**) Local angular velocity, (**c**) Local angular acceleration, (**d**) Superimposing the velocity & acceleration*.*

**Figure 4:**
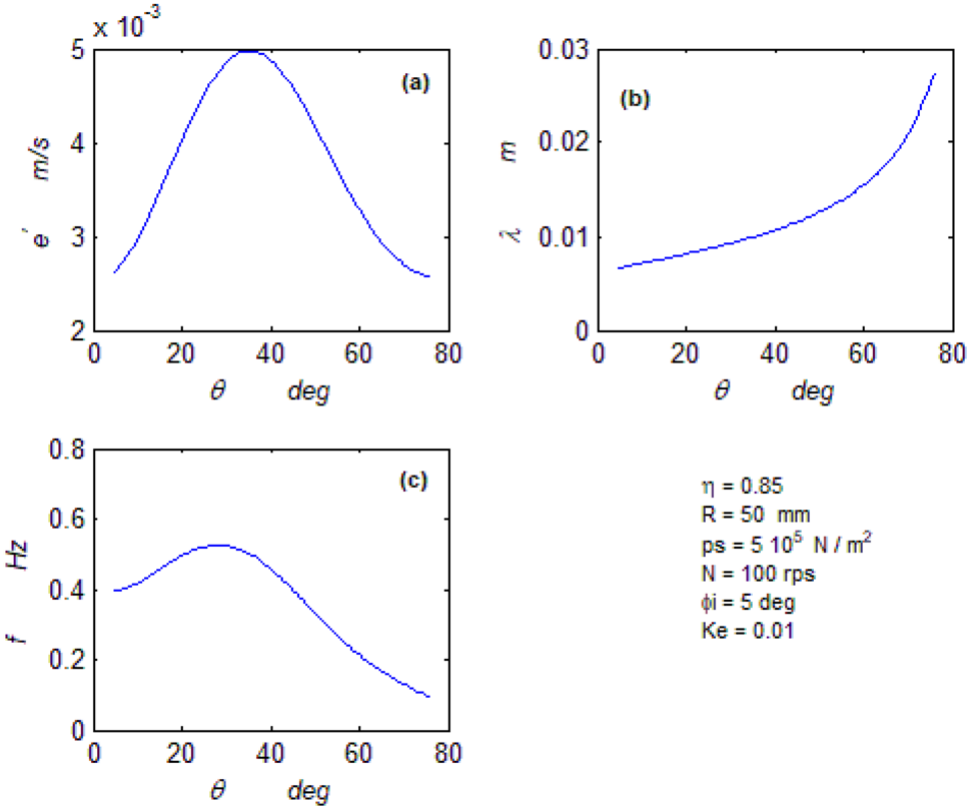
Bearing vibratory factors distribution. (**a**) Linear velocity, (**b**) Wave length, (**c**) Frequency.

**Figure 5:**
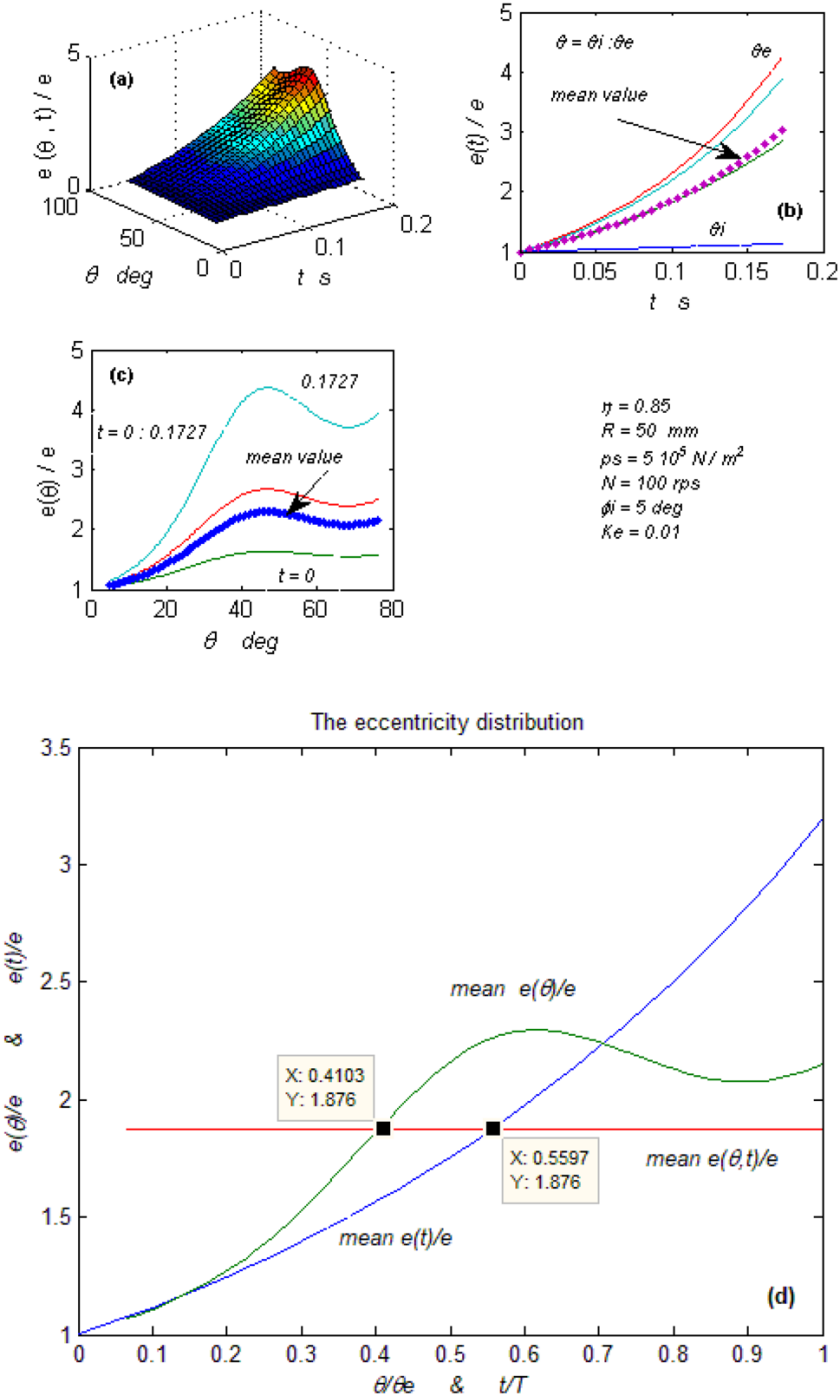
Bearing vibratory factors distribution. (**a**) The normalized eccentricity as a function of (ꝋ) and (t) domains, (**b**) The eccentricity in the time domain at different (ꝋ), (**c**) The eccentricity in the (ꝋ) domain at different time (t), (**d**) Mean eccentricity in (ꝋ) & (t) domains.

**Figure 6:**
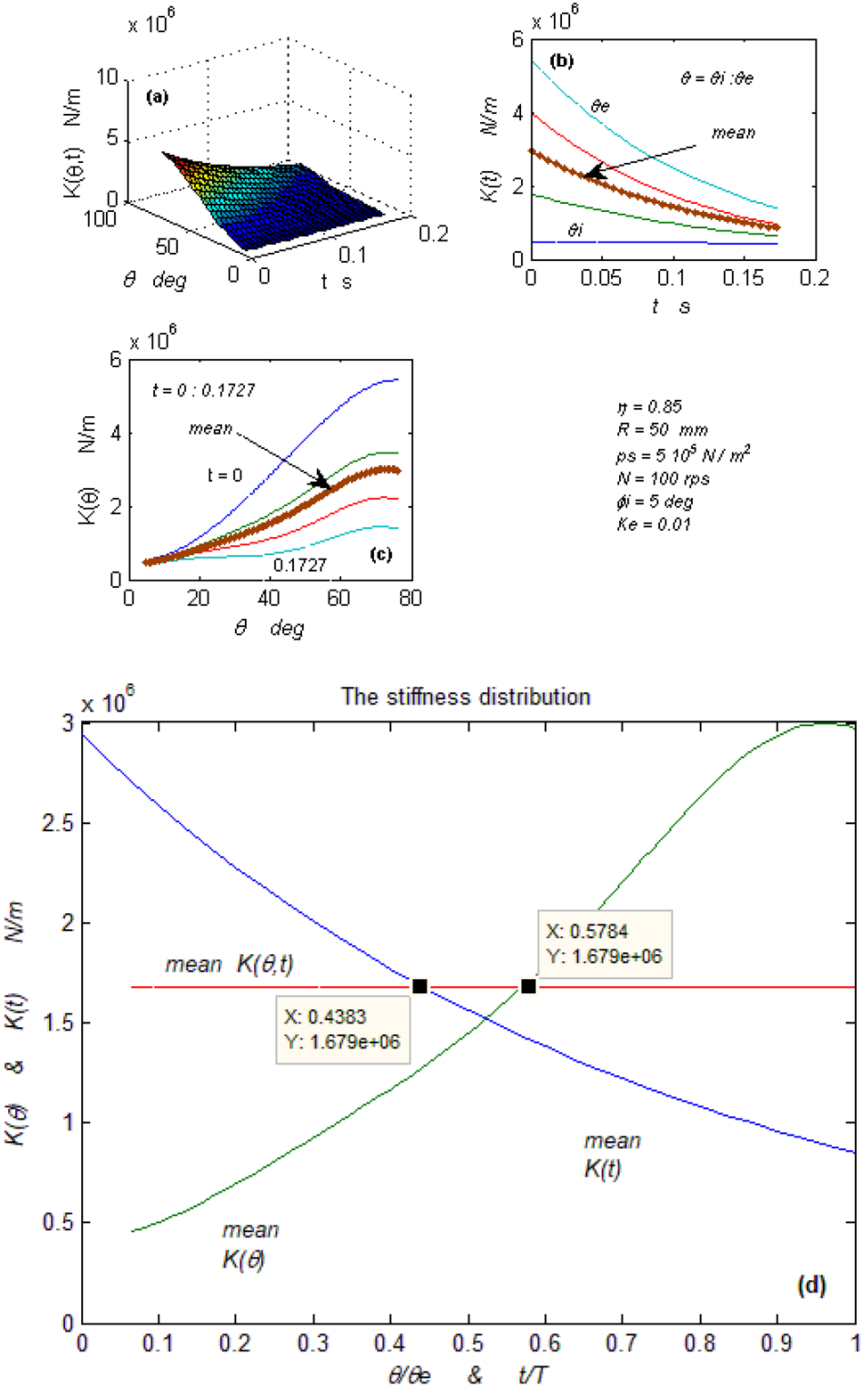
Bearing vibratory factors distribution. (**a**) The stiffness as a function of (ꝋ) and (t) domains, (**b**) The stiffness in the time domain at different (ꝋ), (**c**) The stiffness in the (ꝋ) domain at different time (t), (**d**) The mean stiffness in (ꝋ) & (t) domains.

**Figure 7:**
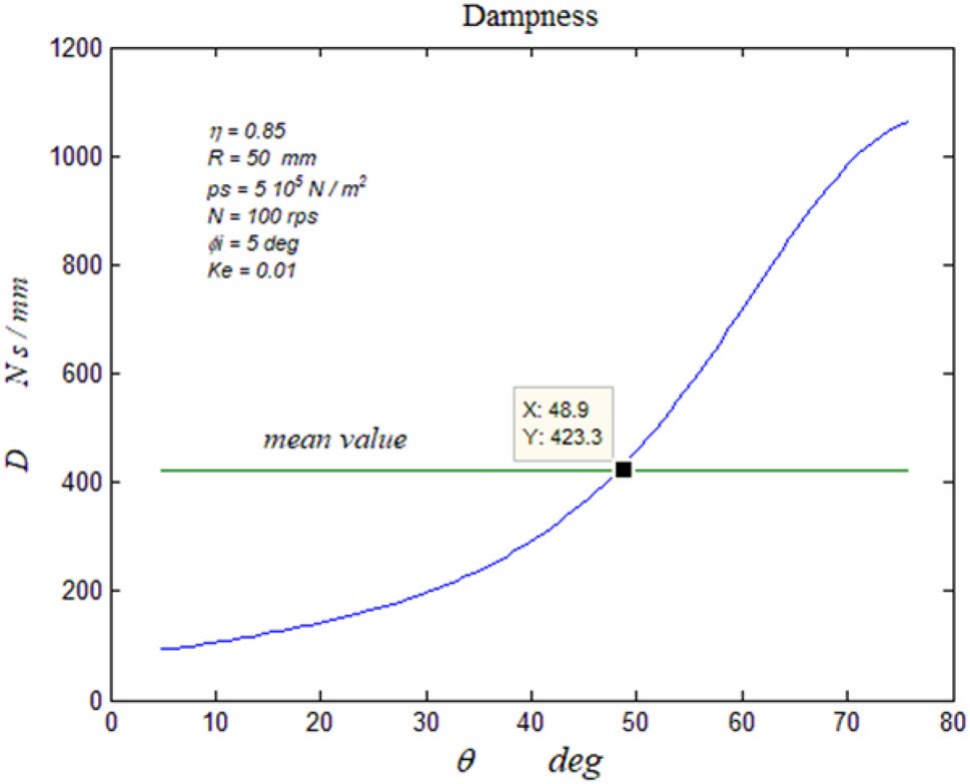
Bearing vibratory factors distribution. The dampness distribution.

**Figure 8:**
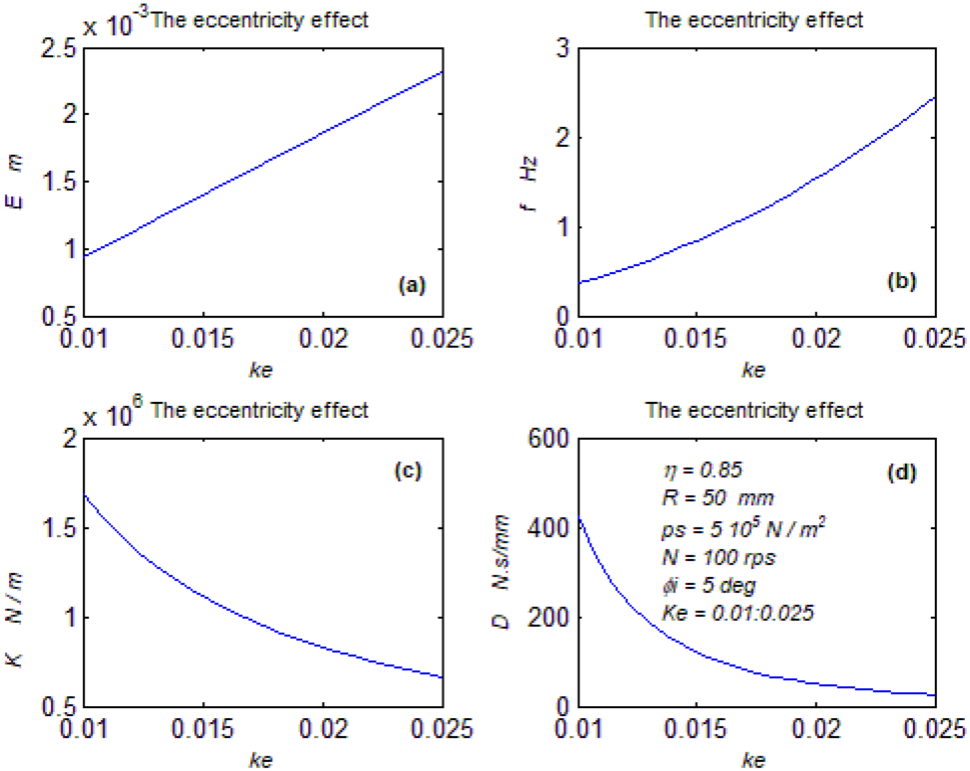
Eccentricity effect on parameters. (**a**) The mean displacement, (**b**) The mean frequency, (**c**) The mean stiffness, (**d**) The dampness.

**Figure 9:**
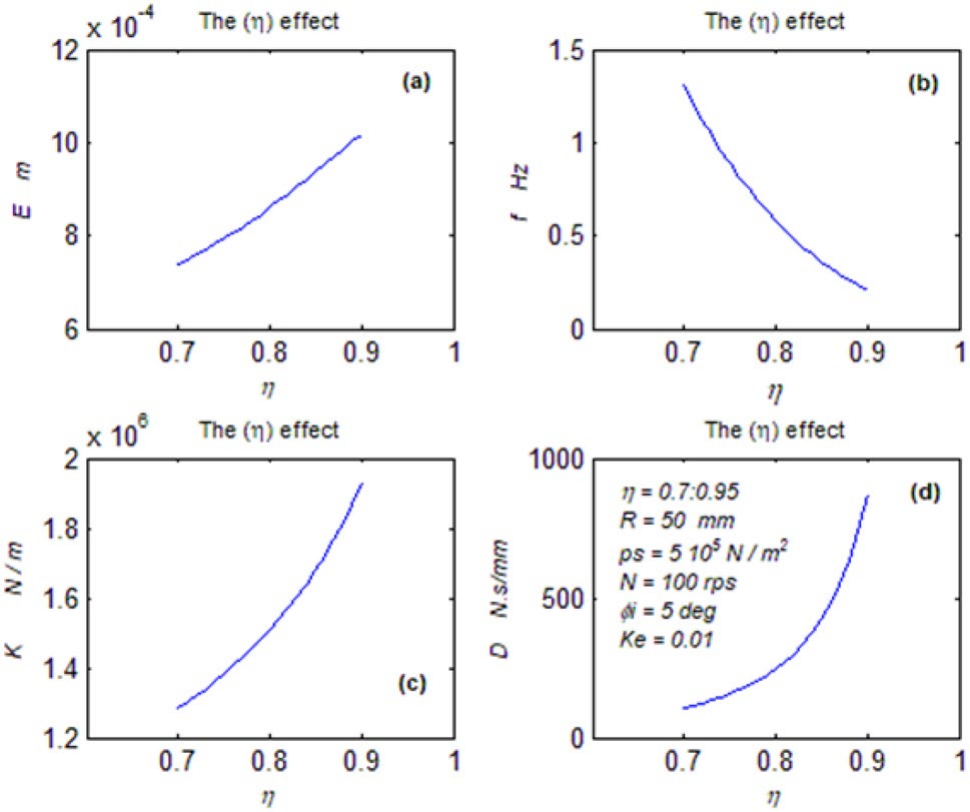
The configuration effect on parameter. (**a**) The mean displacement, (**b**) The mean frequency, (**c**) The mean stiffness, (**d**) The dampness.

**Figure 10:**
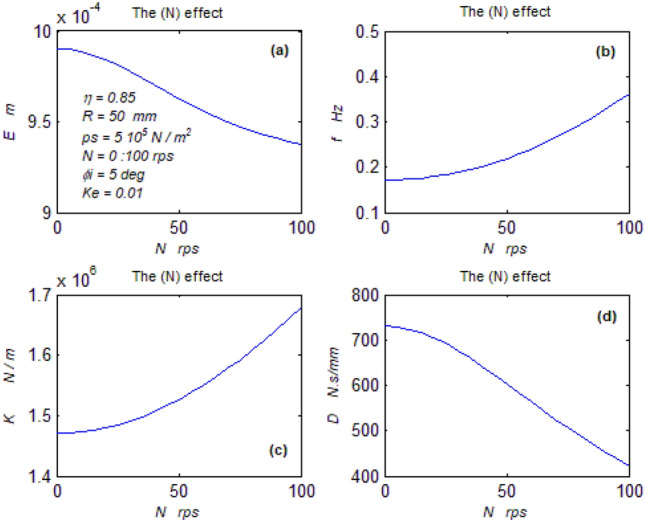
Rotor speed effect on parameters. (**a**) The mean displacement, (**b**) The mean frequency, (**c**) The mean stiffness, (**d**) The dampness.

**Figure 11:**
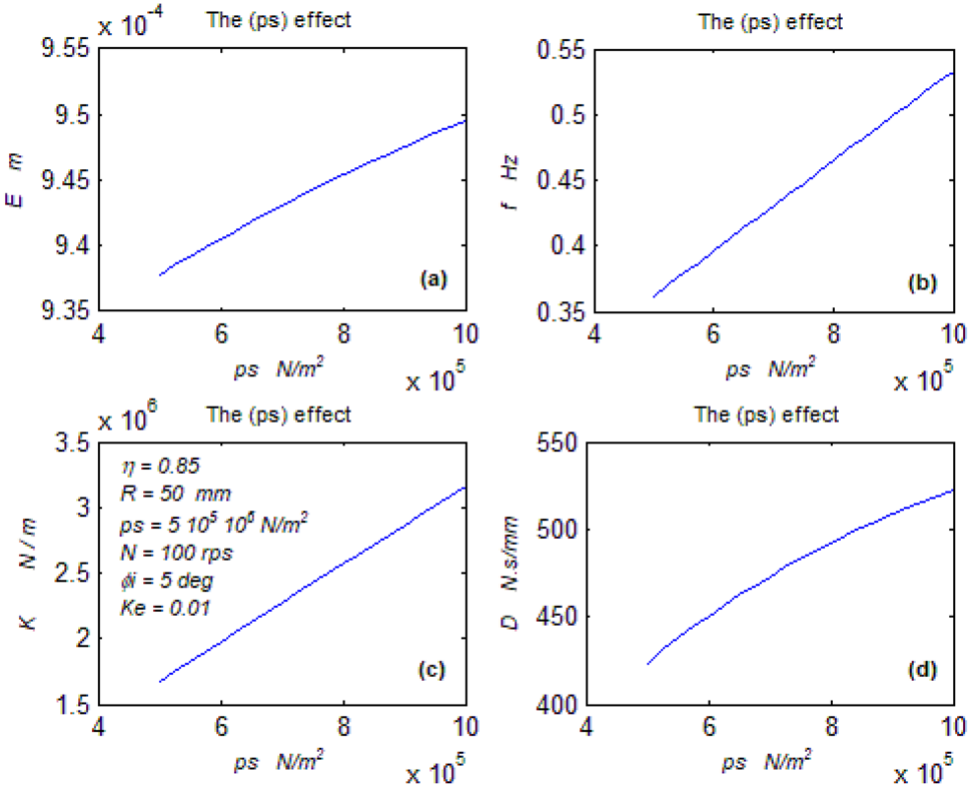
Supply pressure effect on parameter. (**a**) The mean displacement, (**b**) The mean frequency, (**c**) The mean stiffness, (**d**) The dampness.

**Figure 12:**
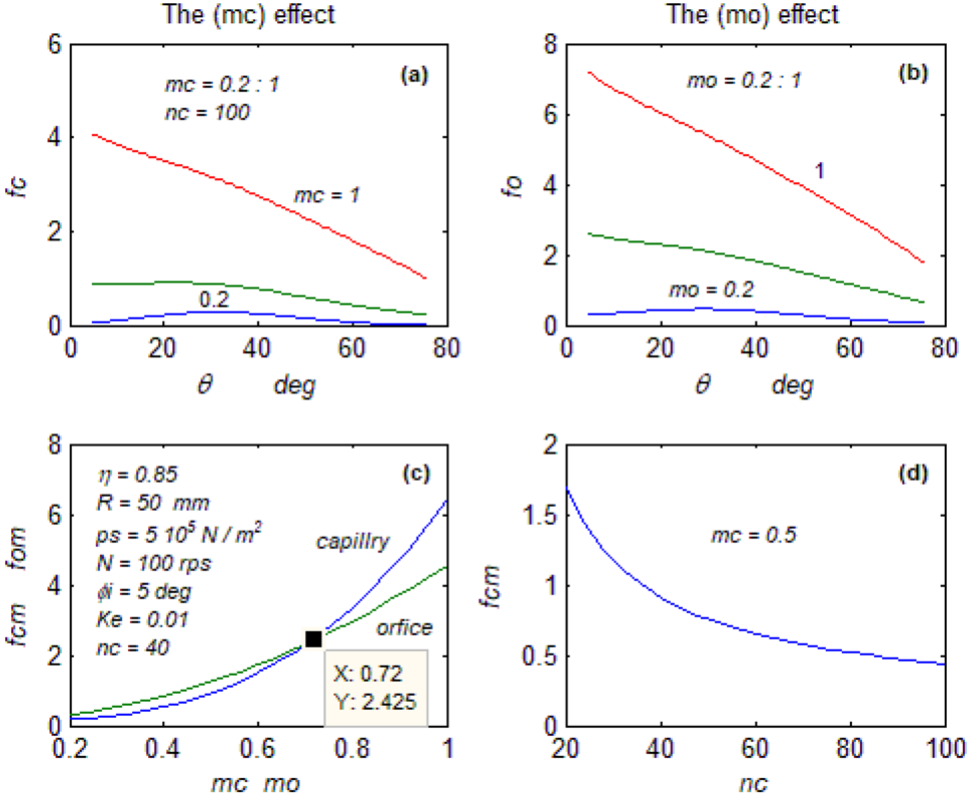
The restrictors effect on the frequency. (**a**) The capillary diameter effect, (**b**) The capillary length effect on the mean frequency, (**c**) The orifice effect, (**d**) The restrictors type effect on the mean frequency.

**Figure 13:**
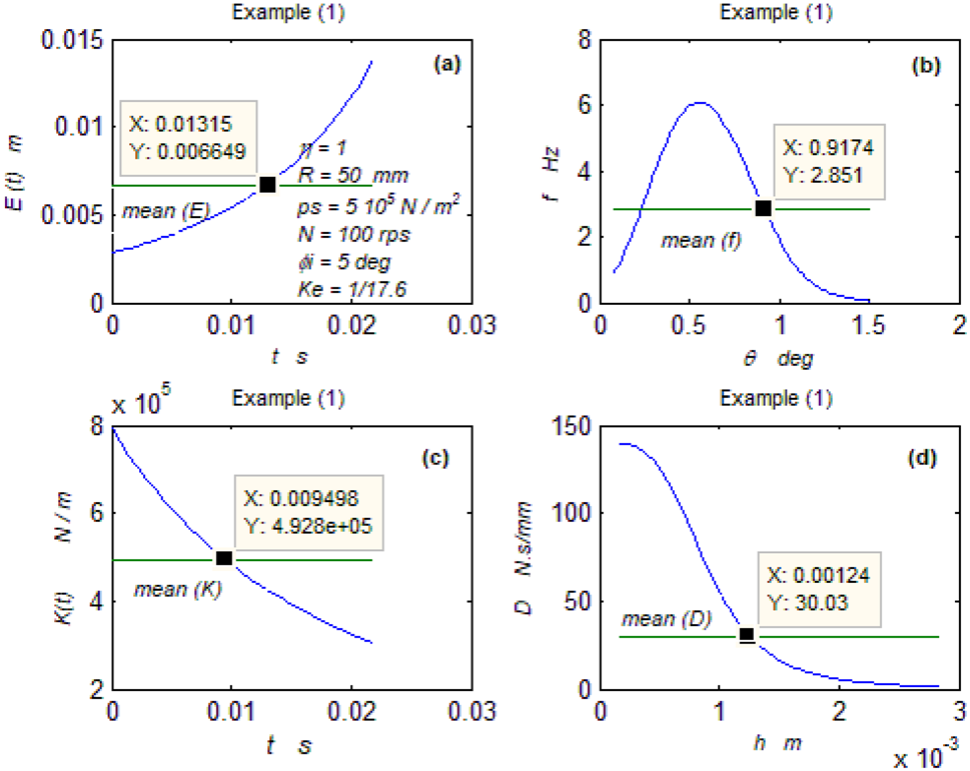
Example (1) parameters. (**a**) The displacement, (**b**) The frequency, (**c**) The stiffness, (**d**) The dampness.

**Figure 14:**
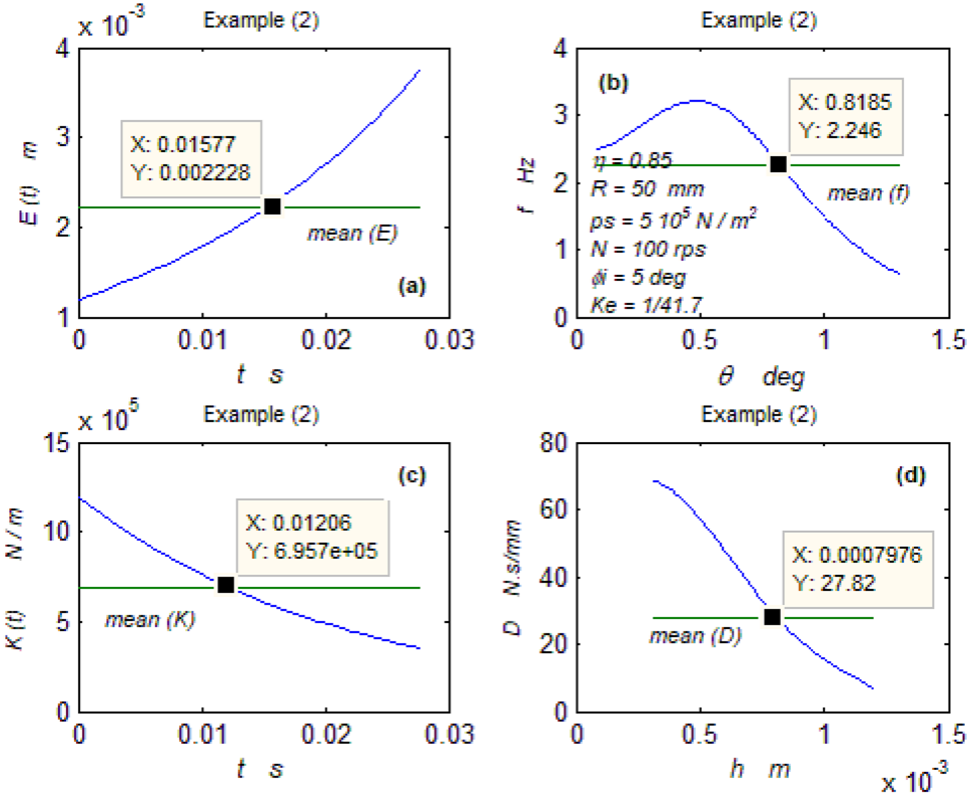
Example (2) parameters. (**a**) The displacement, (**b**) The frequency, (**c**) The stiffness, (**d**) The dampness.

**Figure 15:**
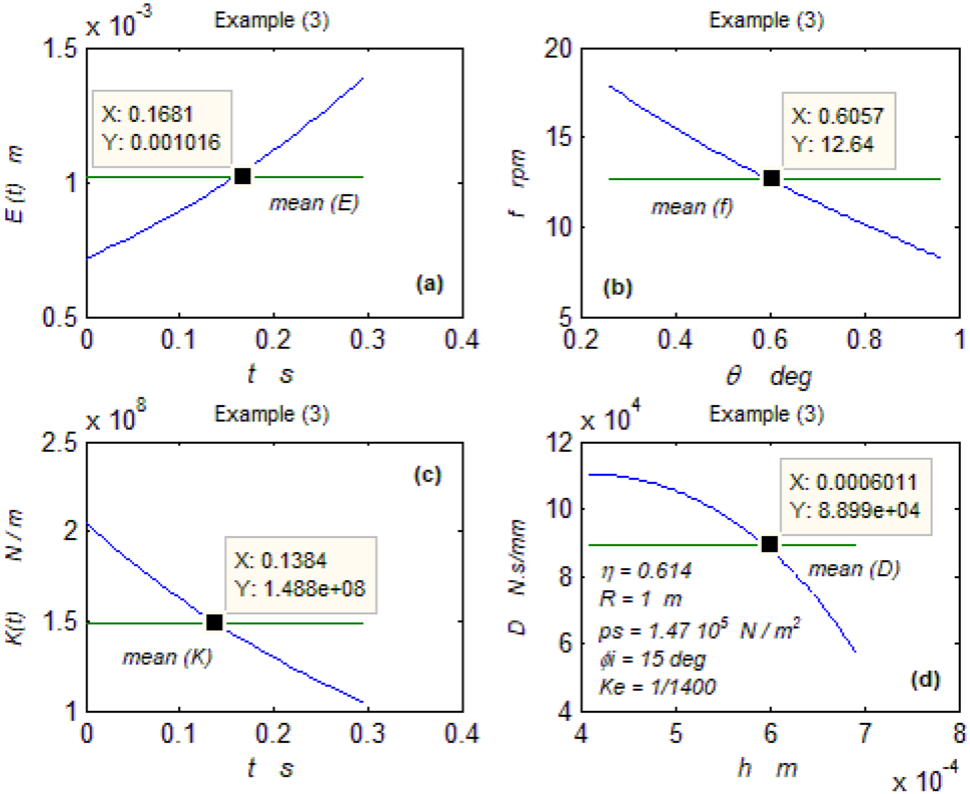
Example (3) parameters. (**a**) The displacement, (**b**) The frequency, (**c**) The stiffness, (**d**) The dampness.

**Figure 16:**
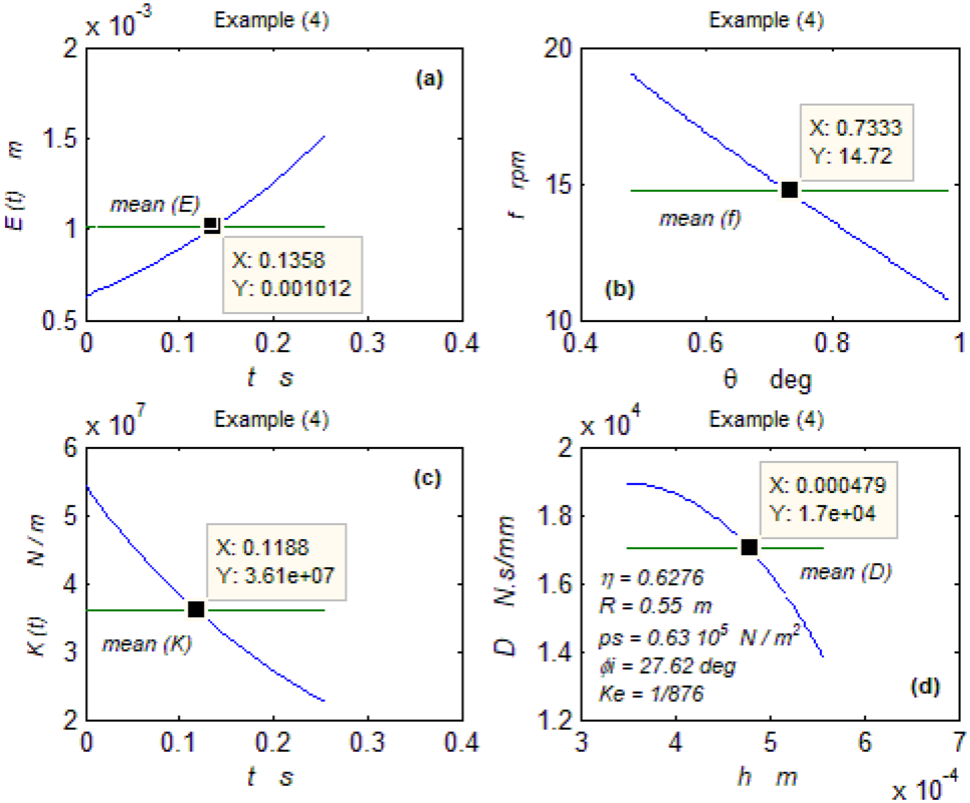
Example (4) parameters. (**a**) The displacement, (**b**) The frequency, (**c**) The stiffness, (**d**) The dampness.

**Figure 17:**
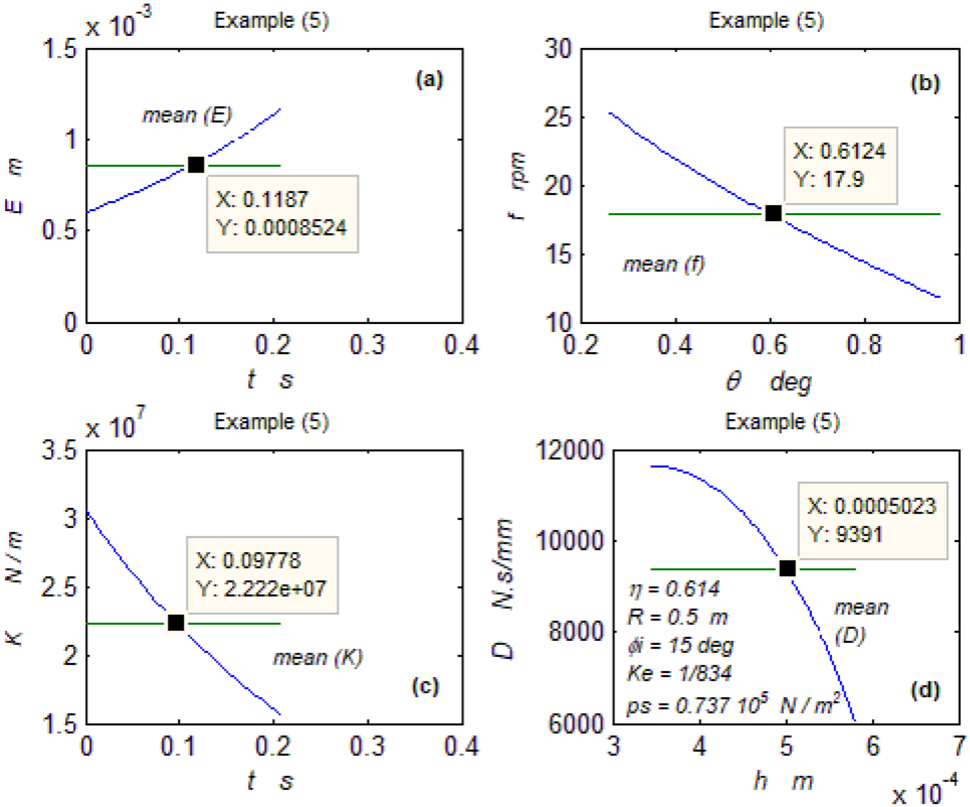
Example (5) parameters. (**a**) The displacement, (**b**) The frequency, (**c**) The stiffness, (**d**) The dampness.

**Figure 18:**
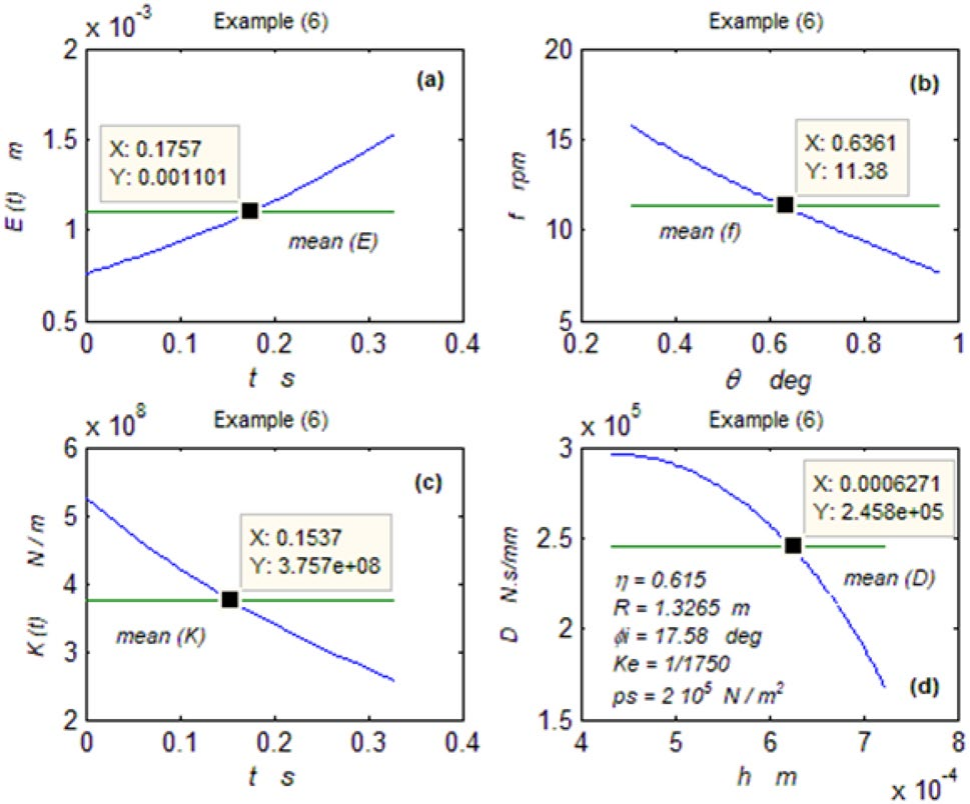
Example (6) parameters. (**a**) The displacement, (**b**) The frequency, (**c**) The stiffness, (**d**) The dampness.

**Figure 19:**
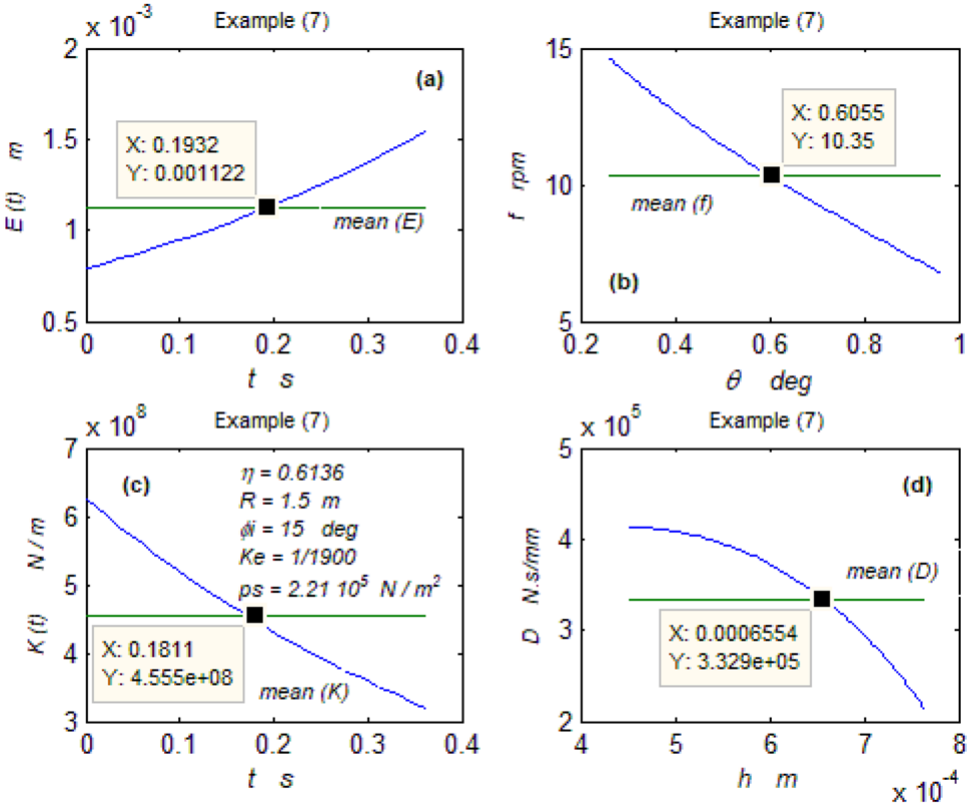
Example (7) parameters. (**a**) The displacement, (**b**) The frequency, (**c**) The stiffness, (**d**) The dampness.

The goal of this paper, as stated previously, is to investigate the vibratory behavior of the fitted thrust spherical hydrostatic bearing. Applying a new method, new and unique formulas have been derived to predict the vibratory bearing characteristics such as the vibration frequency, the stiffness, the dampness etc. The effect of eccentricity, supply pressure, restrictor type and its dimensions, inertia, and seat configuration on the bearing characteristics is studied. Seven cases have been checked to assess the new method’s validity. Figures 3, 4, 5, 6, 7, 8, 9, 10, 11 and 12) show the bearing factors and the behavior under different parameters, while Figs. 13, 14, 15, 16, 17, 18 and 19 show the results of the seven tested examples. Table 1 shows the frequency comparison between the present test results and the previous ones.

**Table 1:** Results of the frequency calculations. Frequency comparison of the examined cases.

Ex. No Fr. Units	1 Hz	2 Hz	3 rpm	4 rpm	5 rpm	6 rpm	7 rpm
Previous	–	–	12.8	13.8	18	11.8	10.5
Present	2.85	2.25	12.64	14.7	17.9	11.38	10.35

## Discussion

The conception of this research arises from the author’s references^6–8, 16, 17^ which point to the vibratory phenomenon of this type of bearings promising to be studied in detail in future separate research. However, a new method is used to study the bearing vibratory behavior avoiding the assumptions of infinitesimal oscillation or disturbance or/and applying the first-order perturbation method as done before. The new method adopted is mainly dependent on bearing hydrodynamics rather than reliance on mechanical analysis as in^2, 11^.

### The new derived formulas

#### Pressure and load capacity

New pressure and load capacity Eqs. (24, 26) have been developed [Appendix A1, A2].

Figure 3a depicts the pressure and load distributions that coincide with that obtained from^4^ and the pressure has the same feature as in^19^ despite the use of non-Newtonian lubricant.

#### The wave parameters

Figure 3b, c, d show the parameters distribution and their mean values. Figure 3b, c express the 1st and 2nd-time derivatives of the variable parameter (ꝋ). The 1st derivative is the angular wave velocity, while the 2nd is the angular wave acceleration. Figure 3d is the superimposing of the angular speed on acceleration, which gives an idea about their relationship, which may not be easily realized from the mathematical equations. The optical examination of Fig. 3d provides a direct impression of the mathematical nature of both curves. It could be mathematically realized that the angular acceleration is a form of the sine function, so the angular velocity is a cosine function (as its time integration) lagged by ninety degrees. Hence, it should be predicted that the displacement (e) in the spatial domain must be in a sinusoidal form (as an integration of the velocity). Their maximum, mean, and minimum values are used in the mathematical calculations, while their positions on the seat could be helpful for industrial inspection.

#### The axial wave velocity

The axial wave velocity is represented by Eq. (17), and its distribution is shown in Fig. 4a. The velocity nonlinearity is revealed by its distribution. The nonlinearity of the velocity produces two equal averages at two different positions on the bearing seat, which could be interpreted mathematically as inconsistency, which exists from the energy difference (Kinetic energy) between these two positions. A mechanical moment is produced by the difference between these two kinetic energy values. This moment causes the bearing rotor to rotate if it has zero degrees of freedom, as in the case of the Kugel ball^2, 11^, and to oscillate if it has one degree of freedom, as in the case of the current bearing. ^2, 11^ calculated this moment mechanically by force analysis and determined the frequency.

The optical examination of the curve reveals that it is of a parabolic form (two equal roots at two different positions), which mathematically means that this axial velocity is a 2nd-degree function. Hence, it should be expected (mathematically) that the displacement (e) in the time domain must be a 3rd-degree function.

#### The wavelength

Equation (18) connects Eqs. (12, 17), yielding the wavelength. Figure 4b depicts its distribution, as well as its mean value and position.

#### The wave frequency

The unique frequency Eq. (12) calculates and predicts the wave frequency (vibration), while Fig. 4c shows the frequency distribution and the mean value, and its position.

#### The wave power

Equation (19) calculates the wave power per unit wavelength to be (0.2517 N/s). The wave power is determined to provide only an idea about the energy which could be lost.

#### The displacement characteristic equation

The unique characteristic Eq. (22) of the rotor's displacement represents mathematically a partial differential equation of an oscillating damped motion. This demonstrates that the bearing eccentricity oscillates, resulting in rotor vibration in the case of a stationary bearing seat (without a damper).

#### The (PDE) solution

Equation (23) is the mathematical solution to the partial differential Eq. (22). The solution demonstrates that displacement is both a time and a spatial function. Figure 5a depicts the synchronous function distributions in both domains. Figure 5b shows the time domain function distributions (at various spatial values) and the distribution of their mean values. Figure 5c shows the spatial domain function distributions (at different time values) and the distribution of their mean values. Figure 5d depicts the superimposing of the mean value distributions in the two domains and the average of each distribution representing the function mean values in the two domains. From the optical examination, it could be realized that Fig. 5b is a 3rd-degree function as predicted before. The function's continuous increase in the time domain leads to the unbalance. Figure 5c looks sinusoidal with severe dampness. This suppresses the function inconsistency in the spatial domain. Hence the function behavior removes the mystery of the resistance to angular displacement property of this type of bearing. The superimposing of the function components in the two domains, Fig. 5d, shows two equal averages at two different positions. This leads to the bearing inconsistency, as was explained previously, by creating a moment that results in rotating or oscillating the bearing rotor depending on its degree of freedom. Hence it could be confirmed that this generated moment is a result exerted due to the potential energy difference between the two averages of the displacement (e) or/and the kinetic energy difference between the two averages of the axial wave velocity (e^o^) as declared before.

#### The bearing stiffness

Equation (27) relates between Eqs. (23, 26) existing the bearing stiffness. The bearing is represented by a loaded vibrating spring in Fig. 2. The stiffness is also a time and spatial function, and Fig. 6a–d represent its distribution as done with displacement (e). Figure 6a shows the synchronous stiffness function distributions in both domains. Figure 6b, c shows that the stiffness in the time domain decreases while increasing in the spatial domain. This makes the bearing more stiffened at the lubricant exit than at the inlet. Figure 6d shows two equal averages of the stiffness function at different times. The difference between the two stiffness averages in the time domain creates internal dampness (stiffness × time = dampness).

This internal dampness reveals the mystery of the self-alignment property characterizing this type of bearing.

#### The bearing dampness

Equation (28) relates Eqs. (17, 26) existing the bearing dampness. Figure 7 shows its distribution where it reaches its maximum at the lubricant exit, which enhances the bearing self-alignment property.

### The bearing behavior

Figures 8, 9, 10 and 11) show the bearing behavior under different factors. The effects of these factors have been applied to the main bearing parameters, the displacement, the frequency, the stiffness, and the dampness. Equations (6, 12, 23, 27, 28) are the main equations that govern this behavior. To ease the grasp of this complicated behavior, attention should be paid to the simple and basic formula used in deriving Eqs. (6).

#### The eccentricity effect on the behavior

As the eccentricity increases ($\theta_{e}$) decreases, in turn, the time (t) must also reduce to keep the (r. h. s) of the formula constant. Hence, the periodic time (T) automatically decreases due to Eq. (6). In turn, the frequency increases, Fig. 8b.

Looking at Eq. (23), it could be realized, despite its complexity that its exponents (K1, K2) increase due to the frequency increase leading to increasing the displacement despite the time reduction Fig. 8a. Also, the eccentricity increase leads to a decrease in the load. In turn, load and displacement increase leads to an increase in the stiffness due to Eq. (27) Fig. 8c.

The reduction in time causes increasing the axial velocity (e^o^). Hence, the decreased load and the increased axial velocity cause decreasing dampness due to Eq. (28), Fig. 8c.

The eccentricity increase severely harms the bearing where the vibration increases and the stiffness and dampness decrease.

#### The configuration effect on the behavior

Figure 9 a-d show the bearing behavior under different configurations, where the seat arc length is expressed by ($\eta$). Referring to the basic formula and the bearing configuration in Fig. 1, it could be seen that the increase in ($\eta$) increases ($\theta_{e}$), in turn, the time (t) to balance the (r. h. s.) of the formula, subsequently the periodic time (T) increases leading to decrease the frequency as could be shown in Fig. 9b. With the increase in *(*$\eta$*)*, the load increases, and with the time increase, the axial velocity (e^o^) decreases; hence from Eq. (28), the dampness increases as in Fig. 9d. With the frequency and time increase, the exponents of Eq. (23) increase leading to the displacement increase, Fig. 9a. With a quick look, the stiffness Fig. 9c shows a contradiction between Eq. (23) and the results. This untrue contradiction arises from the relation between the load and the displacement, where both load and displacement are increasing. However, this means that the stiffness parameter of each case increases as the seat arc length increase, and it is a fact without any doubt about it. The seat arc length increase is crucial for improving bearing behavior. This results in decreased vibration and increased dampness and stiffness.

#### The rotor speed effect on the behavior

Figure 10 a-d show the bearing behavior under different rotational speeds. The rotational speed (N) results in existing inertia, which increases the load^4^.

This case could be treated as the previous item. Like in the case of ($\eta$), the rotational speed (N) increases the load, which means that the (r. h. s) of the basic formula increases. To balance this increase, the (l. h. s) should also increase; hence, because the ($\theta_{e}$) is constant, the time (t) should be decreased; in turn, the periodic time (T) decreases, resulting in the frequency increase, Fig. 10b. With the decrease in the time (t), the axial velocity (e^o^) increases; hence, the dampness decreases as predicted by Eq. (28), where both of load and the axial velocity increase, Fig. 10d.

Figure 10a shows the decrease in the displacement due to the time decrease despite the frequency increase, where the complexity of Eq. (23) doesn’t allow the behavior prediction without calculation. However, in Fig. 10c, the increase in both load and displacement causes the stiffness increase, as explained before. Two important finds were observed; the 1st is that the rotational inertia could severely harm the bearing where the increase in (N) leads to an increase in the frequency and decrease in the dampness, despite the stiffness increase, while the second observation is that the bearing generates vibration in the stationary state (N = 0), this vibration is recognized from the figures and the mathematical calculations confirm it. What is worthy is the ability to internally control and fix the inertia effect by designing the bearing with certain conditions^4^.

#### The supply pressure effect on the behavior

Figure 11a–d show the bearing behavior under different supply pressures.

Figure 11b shows the unpleasant effect of the supply pressure on the bearing frequency. It could be observed from Eqs. (13, 14).

Figure 11c, d show the pleasant effect of the supply pressure on the bearing stiffness and dampness, respectively. It could be realized from Eqs. (27, 28), where the increased pressure increases the load, and at fixed eccentricity (e) and axial velocity (e^o^), the stiffness and dampness increase.

Figure 11a shows the undesired effect of the supply pressure on the bearing displacement. The complicated relation of displacement with pressure could not be easily predicted without calculation, as seen from Eq. (23).

Hence, despite improving the stiffness and dampness, the supply pressure increases the rotor vibration and displacement. Therefore the designer could be guided to compromise between the supply pressure gain and drawback.

#### The restrictor effect on the behavior

Figure 12a–d show the restrictor-type effect on the bearing vibration.

Figure 12a, b show the frequency distribution using the capillary and the orifice at different diameter ratios, the Increasing diameters of the restrictors negatively affect the bearing vibration, and Eqs. (13, 14) predict the frequency increase at increasing the restrictor diameter.

Figure 12c compares the mean frequencies of the capillary and the orifice when varying their diameters; it is clear that at a specific dimension, it could be possible to get the equivalent restrictor which replaces each other.

Figure 12d shows the effect of the capillary length on the frequency, where the increased length highly decreases the frequency, which could be predicted by Eq. (13). Another observation that could be noted from Fig. 12a–c is that the self-restriction bearing^10^, where (m_o_ = 1), generates relatively high vibration.

### Tested examples

Seven cases have been tested. The first two cases were selected from^4, 5^, respectively, and the rest were chosen from^2^.

#### The first two examples

Figures 13 and 14 show that the two bearings have good performances where the frequency and the stiffness are good while the dampness is excellent.

#### The rest of the examples

The five cases are significant because their vibration data could be directly compared with the present ones predicted by the new technique. Figures 15b and 19b and Table 1 below show the coincidence between the results despite their completely different scientific basics. This confirms the validity and reliability of the previous and present techniques.

Despite their different dimensions, the rest of the Figs. 15, 16, 17, 18 and 19 describing the hydrosphere (Kugel ball) show excellent characteristics of high stiffness, high dampness, and low displacement. It may be thought that these characteristics are not significant to be presented here, and the author finds it necessary to get more information about this Kugel ball as a type of hydrostatic thrust spherical bearing.

### The bearing vibration remedy

Despite the importance of this subject, it is out of the scope of this study. It needs a separate detailed investigation which could be the next step in the author’s project for designing an ideal bearing. However, based on the present study, a temporary self-remedy method could be presented by increasing the capillary tube restrictor length, which could highly suppress the bearing vibration.

## Conclusion

As mentioned before, this study is achieved to grasp the vibratory nature of the hydrostatic thrust spherical bearing behavior. The study is an exploring step to enable the researcher, designer, and inspector to put their hands on the bearing problems to find the best solutions. However, the present study offers the following:

### Methodology

A new unique technique based on the Reynolds equation for lubrication for investigating the bearing vibration behavior hydro-dynamically rather than mechanically.

### Mathematics

New and unique mathematical formulas predict the following:

The bearing pressure, load capacity, vibration frequency, rotor displacement, stiffness, dampness and restrictors dimensions.

### Mathematical applications

The above formulas have been applied to a generally selected bearing to study its behavior.

### Studies and analysis

All the previous items and their relations have been studied and analyzed.

### Contentment

For satisfaction, seven different cases designed by several techniques were examined showing excellent results.

### Major finds

- The self-vibration generated by the bearing and calculating its frequency by new unique formulas derived by new method.
- Unique governing characteristic equation predicts the bearing behavior.
- Decoding the self-alignment and the resistance to angular displacement properties that characterize this type of bearing
- Decoding the self-vibration phenomena of the bearing.

### Emphasis

Regardless the technique used in designing this type bearing, attention should be paid to the effect of most important parameters:

- The supply pressure for its negative effect on the bearing vibration.
- The seat arc length for its positive effect on the bearing vibration.
- The rotational speed for its negative effect on the bearing vibration.
- The capillary restrictor length for its positive effect on the bearing vibration.
- The eccentricity for its negative effect on the bearing behavior at all.
- The vibration frequency.

Finally, it could be reliably said that the bearing has been dissected, and the results are impressive.

### Supplementary Information

Supplementary Information.

## Data Availability

The datasets generated and/or analyzed during the current study are available in the Web Link ResearchGate.

## List of symbols

*A*: $$\left( {{{6\mu \,q} \mathord{\left/ {\vphantom {{6\mu \,q} {e^{3} \pi \,p_{i} }}} \right. \kern-0pt} {e^{3} \pi \,p_{i} }}} \right) = Q$$
*A*_*s*_: Bearing projected area ($A_{w} \sin^{2} \theta_{i}$)
*A*_*w*_: Wet projected area ($\pi R^{2}$)
*A*_*v*_: Wave amplitude
*C*: $${{6\,\mu \,q} \mathord{\left/ {\vphantom {{6\,\mu \,q} {p_{i} }}} \right. \kern-0pt} {p_{i} }}$$
*d*_*o*_: Orifice restrictor diameter
*d*_*c*_: Capillary diameter
*d*_*i*_: Bearing inlet orifice diameter
*D*: Dampness
*e* & E: Eccentricity and displacement
$\overline{e}$: 1St time derivative of the eccentricity
$\mathop e\limits^{ = }$: 2Nd time derivative of the eccentricity
*E*: Maximum rotor displacement
*F*(*t*): Function in the time domain
*F*(*ꝋ*): Function in the spatial domain
*f*: Frequency (Hz)
*f*_*cm*_: Capillary mean frequency
*f*_*om*_: Orifice mean frequency
*g*: Gravity acceleration
*h*: Film thickness = *e cos*$\theta$ (*fitted bearing*)
$\overline{h}$: 1St time derivative of the film thickness
*H*: *E*
*K*: Mean stiffness
*K*_e_: ($${e \mathord{\left/ {\vphantom {e R}} \right. \kern-0pt} R}$$)
*K*_c_: Capillary constant
*K*_o_: Orifice restrictor constant
*m*_*c*_: *d*_*c*_/*d*_*i*_
*m*_*o*_: *d*_*o*_/*d*_*i*_
*n*_*c*_: *l*_*c*_/*d*_*c*_
*N*: Rotational speed
*p*_*i*_: Inlet pressure
*p*_*s*_: Supply pressure
*p*_*w*_: Wave power per unit wave length
*P*: $${{\mathop p\limits^{\_\_} } \mathord{\left/ {\vphantom {{\mathop p\limits^{\_\_} } {p_{i} }}} \right. \kern-0pt} {p_{i} }}$$ = Dimensionless pressure
*q*: Lubricant volume flow rate
*Q*: Dimensionless volume flow rate
*R*: Sphere radius
*S*_1_: $${{3\,\rho \,\Omega^{2} R^{2} } \mathord{\left/ {\vphantom {{3\,\rho \,\Omega^{2} R^{2} } {20}}} \right. \kern-0pt} {20}}$$
*S*_2_: $${{3\,\rho \,\Omega^{2} R^{2} } \mathord{\left/ {\vphantom {{3\,\rho \,\Omega^{2} R^{2} } {20\,p_{i} }}} \right. \kern-0pt} {20\,p_{i} }}$$
*t*: Time
*T*: Periodic time
*w*: Load carrying capacity
*W*: Dimensionless load capacity (*w/πR*^*2*^*p*_*i*_)
*β*: $$p_{i} /p_{s}$$
*φ*_*b*_: Seat outer rim angle
*φ*_*i*_: Seat inner rim angle
*θ*: Angle coordinate
$\overline{\theta }$: 1St time derivative of the angle ($\theta$)
$\mathop \theta \limits^{ = }$: 2Nd time derivative of the angle ($\theta$)
*θ*_*i*_: Inlet or recess flow angle
*θ*_*e*_: Outlet flow angle
*ρ*: Oil & water density (867, 1000 N s^2^/m^4^)
*μ*: Oil & water viscosity (0.068, 0.001 N s/m^2^)
*η*: 2*ϕ*_*b*_/*π*
*ω*: *2πf*
Ω: *2πN*
*λ*: Wavelength
