# Supplementary material for: Analytical study of the spherical hydrostatic bearing dynamics through a unique technique
Source: Sci Rep. 2023 Nov 8;13:19364. doi: 10.1038/s41598-023-46296-5 (PMC10632341; doi:10.1038/s41598-023-46296-5)
Supplement: Supplementary file 1 — Supplementary Information. [file 41598_2023_46296_MOESM1_ESM.doc]

**APPENDIX**

***A1- Integration of the pressure partial differential equation***

$$[h^{3}\sin\theta\frac{d \mathop{p}\limits^{--} }{d\theta}]=C+(\frac{3 \rho\Omega^{2} R^{2}}{20}) h^{3} \sin\theta\sin2\theta$$

$$\frac{d \mathop{p}\limits^{--} }{d\theta}=\frac{C}{h^{3}\sin\theta}+(\frac{3 \rho\Omega^{2} R^{2}}{20}) \sin2\theta$$

$$\begin{matrix} \mathop{p}\limits^{--} =\int[\frac{C}{h^{3}\sin\theta}+(\frac{3 \rho\Omega^{2} R^{2}}{20}) \sin2\theta] \partial\theta+B \\ \frac{\mathop{p}\limits^{--} }{p_{i}}=\int[\frac{C}{e^{3}p_{i}\cos^{3}\theta\sin\theta}+(\frac{3 \rho\Omega^{2} R^{2}}{20p_{i}}) \sin2\theta] \partial\theta+B \\ P=\int[\frac{A}{\cos^{3}\theta\sin\theta}+S_{2} \sin2\theta] \partial\theta+B \\ P= \frac{\mathop{p}\limits^{--} }{p_{i}}, A=\frac{C}{e^{3} p_{i}}, S_{2} =(\frac{3 \rho\Omega^{2} R^{2}}{20p_{i}}) \end{matrix}$$

$$P=A\int\frac{1}{\cos^{3}\theta\sin\theta} d\theta+ \int S_{2} \sin2\theta d\theta+B$$

The integration of:

$$\int S_{2} \sin2\theta d\theta=S_{2} \sin^{2}\theta$$

The integration of:

$$A\int\frac{1}{\cos^{3}\theta\sin\theta} d\theta$$

Put:

$$\cos\theta=x$$

$$A\int\frac{1}{\cos^{3}\theta\sin\theta} d\theta=A\int\frac{1}{x^{3}(1-x^{2})} dx$$

Finding the partial fraction as:

$$\frac{1}{x^{3}(1-x^{2})} =\frac{a}{1-x}+\frac{b}{1+x} +\frac{cx^{2}+dx+e}{x^{3}}$$

The coefficients become:

$$a=\frac{1}{2}, b=-\frac{1}{2}, c=1 , d=0 , e=1$$

Hence:

$$\begin{matrix} A\int\frac{1}{\cos^{3}\theta\sin\theta} d\theta=A\int\frac{1}{x^{3}(1-x^{2})} dx \\ =A\int[\frac{1}{2(1-x)} - \frac{1}{2(1+x)}+\frac{x^{2}+1}{x3}] dx \end{matrix}$$

$$\begin{matrix} =A[-\frac{1}{2}\ln(1-x)-\frac{1}{2}\ln(1+x)+\ln(x)-\frac{1}{2x^{2}}] \\ =A[\ln(x)-\ln(1-x^{2})-\frac{1}{2x^{2}}] \\ =A[\ln(\cos\theta)-\ln(\sin^{2}\theta)-\frac{\sec^{2}\theta}{2}] \\ =A[\ln(\cot\theta)-\frac{\sec^{2}\theta}{2}] \end{matrix}$$

Then, the integration becomes:

$$P=A [\ln(\cot\theta)-\frac{\sec^{2}\theta}{2}]+S_{2}\sin^{2}\theta+B$$

Using the boundary conditions:

$$\begin{matrix} P=1 at \theta=\theta_{i} \\ P=0 at \theta=\theta_{e} \end{matrix}$$

Then:

$$\begin{matrix} A=\frac{1-S_{2}(\sin^{2}\theta_{i}-\sin^{2}\theta_{e})}{\ln(\frac{\cot\theta_{i}}{\cot\theta_{e}})-\frac{\sec^{2}\theta_{i}-\sec^{2}\theta_{e}}{2}} \\ B=-A[\ln(\cot\theta_{e})-\frac{\sec^{2}\theta_{e}}{2}]-S_{2}\sin^{2}\theta_{e} \end{matrix}$$

***A2- Integration of the load capacity equation***

$$\begin{matrix} w=\pi R^{2}p_{i}\sin^{2}\theta_{i}+2\pi R^{2}\int p \sin\theta\cos\theta d\theta\\ \frac{w}{\pi R^{2}p_{i}}=\sin^{2}\theta_{i}+2\int\frac{p}{p_{i}} \sin\theta\cos\theta d\theta\\ W=\sin^{2}\theta_{i}+2\int P \sin\theta\cos\theta d\theta\end{matrix}$$

$$\begin{matrix} W=\sin^{2}\theta_{i}+2\int_{\theta_{i}}^{\theta_{e}} \{A [\ln(\cot\theta)-\frac{\sec^{2}\theta}{2}]+S_{2}\sin^{2}\theta+B \} \sin\theta\cos\theta d\theta\\ W=\sin^{2}\theta_{i} \\ +2A\int_{\theta_{i}}^{\theta_{e}} \ln(\cot\theta) \sin\theta\cos\theta d\theta\\ -2A\int_{\theta_{i}}^{\theta_{e}} [\frac{\sec^{2}\theta}{2}]\sin\theta\cos\theta d\theta\end{matrix}$$

$$\begin{matrix} +2\int_{\theta_{i}}^{\theta_{e}} [S_{2}\sin^{2}\theta] \sin\theta\cos\theta d\theta\\ +2B\int_{\theta_{i}}^{\theta_{e}} \sin\theta\cos\theta d\theta\\ W=\sin^{2}\theta_{i}+a_{1}+a_{2}+a_{3}+a_{4} \end{matrix}$$

$$\begin{matrix} a_{1}=2A\int_{\theta_{i}}^{\theta_{e}} \ln(\cot\theta) \sin\theta\cos\theta d\theta\\ =A [\sin^{2}\theta\ln(\cot\theta)-\ln(\cos\theta)+\frac{1}{2}]_{\theta_{i}}^{\theta_{e}} \\ a_{2}=2A\int_{\theta_{i}}^{\theta_{e}} [\frac{\sec^{2}\theta}{2}]\sin\theta\cos\theta\partial\theta=-A [\ln(\cos\theta)]_{\theta_{i}}^{\theta_{e}} \\ a_{3}=2\int_{\theta_{i}}^{\theta_{e}} [S_{2}\sin^{2}\theta] \sin\theta\cos\theta\partial\theta=\frac{S_{2}}{2} [ \sin^{4}\theta]_{\theta_{i}}^{\theta_{e}} \end{matrix}$$

***A3. Useful relations***

***A3.1 Stiffness and damping***

$$Stiffness x time=Damping$$

$$\begin{matrix} K= \Delta W/\Delta X \\ D=\Delta W/\Delta V \end{matrix}$$

$$\begin{matrix} K=(\Delta W/\Delta X) x \Delta t =\Delta W x (\Delta t/\Delta X) \\ = (\Delta W/\Delta V) =D \\ K x t = D \end{matrix}$$

*V = Velocity*

***A3.2 Kinetic energy and moment***

Difference of kinetic energy = Moment

$$\begin{matrix} \Delta(KE)= (\Delta W/2 g) x \Delta v^{2} \\ Units \\ \frac{N . s^{2}}{m} x \frac{m^{2}}{s^{2}} = N . m \end{matrix}$$

***A3.3 Potential energy and moment***

Difference of Potential energy = Moment

$$\begin{matrix} \Delta(PE)= \Delta W x \Delta Y \\ Units \\ \Delta(PE)= N . m \end{matrix}$$
